# The Role of Telemedicine for Psychological Support for Oncological Patients Who Have Received Radiotherapy

**DOI:** 10.3390/curroncol30050390

**Published:** 2023-05-19

**Authors:** Morena Caliandro, Roberta Carbonara, Alessia Surgo, Maria Paola Ciliberti, Fiorella Cristina Di Guglielmo, Ilaria Bonaparte, Eleonora Paulicelli, Fabiana Gregucci, Angela Turchiano, Alba Fiorentino

**Affiliations:** 1 Radiation Oncology Department, General Regional Hospital F. Miulli, Strada Provinciale 127, Acquaviva delle Fonti, 70021 Bari, Italyflodigu@libero.it (F.C.D.G.);; 2 Department of Medicine and Surgery, LUM University, Casamassima, 70010 Bari, Italy

**Keywords:** radiotherapy, oncology, psychological assessment, tele-consult, telemedicine

## Abstract

AIM: In our radiation departments, all patients received psycho-oncological support during RT and during follow-up. Based on the latter, the aim of this retrospective analysis was to evaluate the role of tele-visits and in-person psychological support for cancer patients after RT, and to report a descriptive analysis pointing out the needs of psychosocial intervention in a radiation department during radiation treatment. METHODS: According to our institutional care management, all patients receiving RT were prospectively enrolled to receive charge-free assessment of their cognitive, emotional and physical states and psycho-oncological support during treatment. For the whole population who accepted the psychological support during RT, a descriptive analysis was reported. For all patients who agreed to be followed up by a psycho-oncologist, at the end of RT, a retrospective analysis was conducted to evaluate the differences between tele-consultations (video-call or telephone) and on-site psychological visits. Patients were followed up by on-site psychological visit (Group-OS) or tele-consult (Group-TC) visit. For each group, to evaluate anxiety, depression and distress, the Hospital Anxiety Depression Scale (HADS), Distress Thermometer and Brief COPE (BC) were used. RESULTS: From July 2019 to June 2022, 1145 cases were evaluated during RT with structured psycho-oncological interviews for a median of 3 sessions (range 2–5). During their first psycho-oncological interview, all the 1145 patients experienced the assessment of anxiety, depression and distress levels with the following results: concerning the HADS-A scale, 50% of cases (574 patients) reported a pathological score ≥8; concerning the HADS-D scale, 30% of cases (340 patients) reported a pathological score ≥8, concerning the DT scale, 60% (687 patients) reported a pathological score ≥4. Eighty-two patients were evaluated after RT: 30 in the Group-OS and 52 in the Group-TC. During follow-up, a median of 8 meetings (range 4–28) were performed. Comparing psychological data at baseline (beginning of RT) and at the last follow-up, in the entire population, a significant improvement in terms of HADS-A, global HADS and BC was shown (*p* 0.04; *p* 0.05; and *p* 0.0008, respectively). Compared to baseline, statistically significant differences were observed between the two groups in terms of anxiety in favor of on-site visit: Group-OS reported a better anxiety score compared with Group-TC. In each group, a statistical improvement was observed in BC (*p* 0.01). CONCLUSION: The study revealed optimal compliance to tele-visit psychological support, even if the anxiety could be better controlled when patients were followed up on-site. However, rigorous research on this topic is needed.

## 1. Introduction

Receiving a cancer diagnosis leads to the development of several psychological dynamics: fear of dying, uncertainty, loss of control, change in interpersonal relationships and change in self-image. For these patients, the psychological structure often represents “baggage” during cancer treatments. Surgery, chemotherapy and radiotherapy (RT) are the main treatment weapons in the armamentarium of the oncological battle. These therapies could be stressful for patients (fear of oncological pathway, fear of side effects, fear of pain, fear of death); however, they have a “life-saving” purpose.

In the present context, during the last few years, SARS-CoV-2 infection has had a significant impact on everyone’s daily life: higher levels of perceived stress were associated with the fear of infection in the whole population, as suggested by recent studies [1]. Furthermore, the lives of oncological patients received additional stress due to the COVID-19 pandemic [2]. In fact, they experienced additional conflicting emotional moods due to the needs of receiving life-saving treatments and the necessity to “stay at home” for fear of exposing themselves to the contagion risk [2,3,4,5].

Moreover, the loneliness that affects all oncological patients added to the social distancing procedures/restrictions in access to care may increase patient fears and concerns about receiving cancer treatments and disease recurrence [6,7,8]. In the management of the relationship between stressful events and disease, the possibility of receiving dedicated support, both in the intimate and extended environment, is a relevant aspect. However, as the pattern and duration of stressors might vary, the responses of each individual could also be significantly variable. They depend on cognitive, neurobiological and emotional processes, such as the style of attachment or the coping performed in response to stress [9]. The differences in the latter responses among patients contribute to higher or lower rates and severity of psychological and/or physical symptoms: for the cancer population, depression occurs in 9–30% and anxiety in 9–50% [9,10,11,12,13,14,15,16,17]. During the COVID-19 pandemic, a high level of stress, about 30%, was reported in the general population, while in the cancer population, it is slightly increased [2]. In addition, the Impact of Event Scale-revised (IES-R) total scores showed a strong state of stress: these findings indicate that during this pandemic, oncological patients experienced a clinically meaningful level of stress that could be comparable to post-traumatic stress disorder [15,17]. In particular, as noted in a recent systematic review, the prevalence rates for COVID-19-related anxiety and depression were 31.9% and 33.7%, respectively, while in the oncological population, they occurred up to 31% and 36%, respectively [2,15,16,17]. For these reasons, the role of psychological support for cancer patients is emerging as a priority.

RT provides significant survival benefit for patients affected by cancer [18], and the benefit of psychological support for oncological patients has been demonstrated [8] both during radiation therapy and during follow-up.

Due to the COVID-19 pandemic, the use of telemedicine (video or telephone visits), in order to avoid the unnecessary exposure risks in an ambulatory hospital, experienced unexpected growth. For oncological patients, psychological meetings could be difficult to organize due to the various disease commitments (chemotherapy appointments, medical visits, diagnostic procedures, and so on). Based on COVID-19 experiences, the expansion of telemedicine for psychological assessment could endure due to the potential benefit for both oncological patients and the healthcare system [19]. Telemedicine may provide consistent advantages over in-person care, especially in chronic patients such as oncological ones, which require frequent psychological assessment and support. Surely, if patients do not have basic technology skills and do not feel comfortable using technologies (phone, computer, and so on), it is difficult to receive any benefit from telemedicine. This may be challenging for some older patients (due to inexperience/lack of access to technology) and for patients with cognitive disability [19].

Many studies pre- and post-COVID-19 showed the benefit of telemedicine in cancer patients, but few data are reported on psychological tele-visits, especially in patients who underwent radiation treatment [20,21,22,23,24,25,26,27,28,29,30].

In our radiation department, all patients received psycho-oncological support during RT and during follow-up. Based on the latter, the aim of this retrospective analysis was to evaluate the role of tele-visits and in-person psychological support for cancer patients after RT, and to report a descriptive analysis pointing out the needs of psychosocial intervention in a radiation department during radiation treatment.

## 2. Materials and Methods

From June 2019 to June 2022, according to our institutional care management, all patients receiving radiation therapy were prospectively enrolled to receive charge-free assessment of their cognitive, emotional and physical states and psycho-oncological support during treatment.

For the whole population who accepted the psychological support during RT, a descriptive analysis was reported.

For all patients who accepted to be followed up by a psycho-oncologist, tele-consultations (video-call or telephone) (TC group) or on-site psychological visits (OS Group) were proposed.

Inclusion in the TC group or OS group was based on patients’ preferences. Inclusion criteria were as follows: patients aged more than 18 years old; diagnosis of cancer; treated with a long course of RT (more than 5 fractions); signed consent form; and psychological assessment and evaluation during radiation therapy and during follow-up (minimum 2 meetings after RT). Exclusion criteria were as follows: no signed consent form; no evaluation during follow-up; age less than 18 or more than 80 years old.

The aims of this analysis were to describe (in terms of anxiety, depression, coping and post-traumatic stress disorder) the whole population, who received psychological support during radiation therapy, and to evaluate the differences in terms of psychological results between tele-visits or in-person visits for the sub-population who were followed up after RT.

For those who adhered to the psycho-oncological support program, the first interview (pre-RT/baseline) and all subsequent psychological meetings were structured as follows by a psycho-oncologist:

1. A descriptive collection of personality features, psychosocial elements and cognitive and emotional processing capacity regarding the event, paying attention to the patient’s history.
2. An evaluation of anxiety and depression with Hospital Anxiety Depression Scale (HADS) [31,32], which is a four-point scale (from 0 to 3) consisting of 14 questions to which the patients had to respond, referring to their symptoms during the previous week. The HADS scale is divided into 2 subscales which investigate anxiety (HADS-A) and depression (HADS-D), respectively. Seven of the 14 elements of the HADS scale correspond to the HADS-A subscale and the remaining 7 to the HADS-D subscale, ranging from 0 (no discomfort) to 21 (extreme discomfort). Scores of 8 or more in both subscales indicate the presence of a disorder.
3. An evaluation of distress using the Distress Thermometer (DT) [33]. It is a single-item instrument, consisting of a visual analog scale and represented by a thermometer ranging from 0 (no discomfort) to 10 (extreme discomfort). A score of 4 or more indicates the presence of a disorder.
4. An assessment of the varying coping strategies used by patients in response to stress by the Brief COPE (BC) [34,35]. It is an instrument composed of 14 scales for a total of 28 items (positive restructuring, distracting attention, expression, use of instrumental support, operational coping, denial, religion, humor, behavioral disengagement, use of emotional support, substance use, acceptance, planning, self-accusation).
5. An evaluation of post-traumatic stress symptoms using the Impact of Event Scale-revised (IES-R) [36]. The IES-R is a 22-item self-report measure (for *DSM-IV*) that assesses subjective distress caused by traumatic events. It is a revised version of the older version; the IES-R contains 7 additional items related to the hyperarousal symptoms of PTSD (post-traumatic stress disorder), which were not included in the original IES. Respondents are asked to identify a specific stressful life event and then indicate how much they were distressed or bothered during the previous seven days by each “difficulty” listed. Items are rated on a 5-point scale ranging from 0 (“not at all”) to 4 (“extremely”). The IES-R yields a total score (ranging from 0 to 88); significant symptoms were defined by a score of more than 33.

During radiation therapy and follow-up, easy implementations, including mindfulness-based stress reduction techniques, were performed to manage stress and pain perception, to reduce physical symptoms and to improve mood and sleep quality [8,35]. These psycho-therapeutic approaches were developed in individual sessions and/or in small group sessions (only during RT), based on the inclination of the patient [8].

### Statistical Analysis

A statistical analysis was carried out to detect differences between study groups, using a Mann–Whitney test for quantitative variables to evaluate differences in median, while t-tests or Chi-square tests were used for qualitative variables to test differences in proportion. A p-value equal or less than 0.05 was considered statistically significant.

## 3. Results

### 3.1. Overall Population Who Received Psychological Assessment during RT

From July 2019 to June 2022, 2730 oncological patients underwent RT in our radiation oncology department. Of these, 2290 (84%) agreed to participate in a psycho-oncological support program, receiving at least a first evaluation. The median age was 60 years (range 20–78). There were 1671 females (73%) and 619 males (27%). In terms of anxiety, depression and distress, for the entire study population during RT, the median HADS-A was 8 (range 1–19), the median HADS-D was 5 (range 1–18) and the median DT was 5 (range 0–10).

Excluding patients treated with a short course of RT (1–5 fractions) who received only one psychological evaluation, the remaining 1145 cases were evaluated during RT with structured psycho-oncological interviews for a median of 3 sessions (range 2–5). For the latter population, the median RT fractions was 25 (range 15–33) and the most common histologies were breast and gynecological cancer.

During their first psycho-oncological interview, all the 1145 patients experienced the assessment of anxiety, depression and distress levels, reporting the following results: concerning the HADS-A scale, 50% of cases (574 patients) reported a pathological score ≥8; concerning the HADS-D scale, 30% of cases (340 patients) reported a pathological score ≥8; concerning the DT scale, 60% (687 patients) reported a pathological score ≥4.

All patients completed their radiation treatment without delays, reporting a reduction in RT anxiety during the interviews and a “feeling of welcome” at the end of RT.

#### Population Followed after RT: Tele-Visit Versus in-Person Evaluation

From July 2019 to June 2022, only 82 patients out 1145 (7.2%) were followed up after RT: 30 patients with on-site consult (Group OS) and 52 patients with tele-consult (Group TC). The main clinical and physiological characteristics are reported in Table 1. Sixty-one patients (74.4%) were female and 21 (25.6%) were male; the median age was 50 years old (range 18–75 years old). The breast cancer (39 cases, 47.5%), gynecological cancer (8 cases, 9.7%), brain tumor (12 cases, 14.6%) and prostate cancer (6 cases, 7.3%) were the most represented histopathologies that affected patients. During RT, all patients received a median of 4 psychological visits, while during follow-up, they received a median of 8 meetings (1 or 2 meeting per week, range 4–28), without significant difference between the two groups.

**Figure 1:**
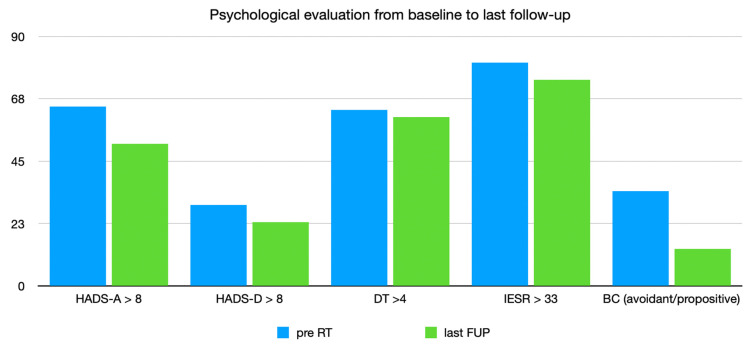
Psychological evaluation pre-RT and at last follow-up for entire population.

For the whole population of 82 patients, from baseline to the last follow-up, a significant improvement in terms of HADS-A, global HADS and BC was shown (*p* 0.04; *p* 0.05; *p* 0.0008, respectively). Figure 1 shows the differences in terms of each scale (and relative pathological value) between baseline and last follow-up.

Regarding Group-OS, the following ratings of anxiety, depression and distress were reported, comparing baseline to last follow-up evaluation, respectively: regarding the HADS-A scale, a score of 9 (range 4–16) versus 8 (4–14); regarding the HADS-D scale, a score of 6 (range 2–14) versus 5.5 (2–11); regarding the DT scale, 20 patients (66.7%) versus 19 patients (63.3%) reported a pathological score ≥ 4; regarding the BC scale, 10 patients (33.3%) versus 3 patients (10%) reported an avoidant behavior; regarding the IES-R scale, a score of 51 (range 14–83) versus 43 (range 11–88).

Regarding Group-TC, the following ratings of anxiety, depression and distress were reported, comparing baseline to last follow-up evaluation, respectively: regarding the HADS-A scale, a score of 8.5 (range 3–16) versus 7.7 (3–12); regarding the HADS-D scale, a score of 7 (range 2–14) versus 6 (2–12); regarding the DT scale, 32 patients (61.5%) versus 31 patients (59.6%) reported a pathological score ≥ 4; regarding the BC scale, 18 patients (34.6%) versus 8 patients (15.4%) reported an avoidant behavior; regarding the IES-R scale, a score of 51 (range 12–88) versus 46.5 (range 11–80).

**Figure 2:**
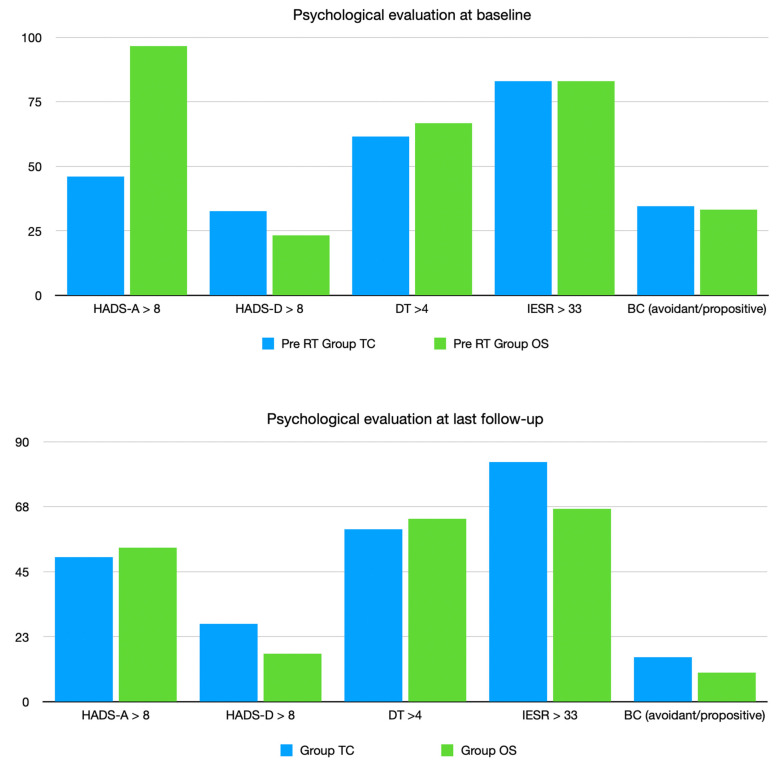
Psychological evaluation pre-RT and at last follow up for each group (tele-consult and on-site).

In general, with respect to baseline, statistically significant differences were observed between the two groups in terms of anxiety (HADS, HADS-A: *p* 0.00002 and 0.03) in favor of on-site visits. In each group, a statistical improvement was observed in BC (*p* 0.01), score ≥ 4. Figure 2 shows, for each scale used, the percentage of patients with pathological score at baseline and at last follow-up for each group (tele-consult and on-site).

## 4. Discussion

The aims of the present analysis were to describe the role of psychological support during radiation therapy in oncological patients, and to evaluate the difference in terms of the emotional states of oncological patients who were followed up by tele-consults or in-person visits, to define the role of telemedicine in this setting.

In order to reduce the number of on-site visits, telemedicine has grown during the pandemic, but few studies in the scientific literature have assessed its employment [20,21,22,23,24,25,26,27,28,29,30].

Telemedicine can improve the burden on the healthcare system by reducing appointments, especially with regard to patients with physical disabilities and dependency on caregivers for better quality of life. Moreover, at the base of the success of telemedicine, there is the need for patients to be able and willing to use technology. A recent evaluation on 15 elderly patients with early breast cancer showed that 13 participants reported a preference for a hybrid care model: telemedicine care plus on-site visits, rather than in-person care alone [19]. The in-person appointment is preferred for 2 reasons: the comfort with familiar face-to-face interactions, and to have a physical exam. In this study, patients preferred in-person appointments during the post-primary treatment phase, while they preferred telemedicine when relationships were well-established and the patients were in follow-up.

To our knowledge, in fact, no substantial data evaluating the changes in emotional states of cancer patients treated with radiation therapy are reported.

Receiving a cancer diagnosis is associated with higher levels of perceived stress; therefore, at the beginning of RT treatment, psychological support was offered to all patients.

In analyzing how many patients adhered to our therapeutic protocol, it emerged that a significant increase in psychological requests were reported during the COVID-19 pandemic in 2020. Particularly, with respect to the July to December 2019 period (pre-COVID), there was a significant increase in patients requesting support (13 patients per month pre COVID-19 period vs. 60 patients per month post-COVID period; *p* < 0.0001). Regarding anxiety and depression for all populations of the study, based on two scales, the data showed that anxiety was present in roughly 50% of patients with a median HADS-A of 8, while only about 30% of patients suffered depression (median HADS-D of 5). These data are in line with other publications [2,13,14,15,16,17].

Moreover, some data in the literature showed that the word “radiation” is often associated with a negative connotation based on media information or a secondhand experience. This produces a high grade of anxiety in patients who are going to receive RT. Gillan et al. conducted a multiphase survey to define the perceptions of radiation in the general population (phase I) and, in phase II, to define the perceptions of RT in patients who received RT [37]. The latter study assessed that in the phase I survey, the public perceptions of radiation were usually negative. In phase II of this analysis, toxic events during and after RT were reported as concerns, such as misperceptions about infertily due to radiation and becoming radioactive. In conclusion, in Canada, many patients refused RT only for fear or misinformation about risks, or they accepted it, but with a high grade of anxiety.

For this reason, the psychological support during active therapy, especially RT, was useful. In fact, all patients completed their treatment without delays, reporting a reduction in RT anxiety during the interviews and a “feeling of welcome” at the end of treatment.

Regarding the aim of establishing the difference between on-site and telemedicine, 82 patients accepted our psychological support by a psycho-oncologist after RT: 52 out 82 (63.4%) wanted to be followed by a tele-visit (video call or telephone). As reported by Buse et al., and also for our population, COVID-19, physical disability, and transportation burdens were the most common factors for tele-visit preference. The median number of visits during follow-up was 8 (range 4–28), with no difference between the two groups.

Anxiety and depression have expression throughout the body. During the psychological interview of all patients, it was possible to reduce the levels of distress and somatic disorders, including fatigue, sleep disorders, gastritis, cognitive ruminations and risk behaviors. Cognitive-behavioral techniques for processing destabilizing events, from diagnosis to related treatments, and relaxation techniques for the management of anxiety and pain, were used to obtain anxiety reduction. Our data showed that the psychological support during and after RT treatment reported an improvement in anxiety and depression, by activating a propositive behavior (BC) for a better quality of life perception.

In terms of depression and post-traumatic stress, no improvements were documented. This is probably due to the emotional state of oncological patients after treatment and during follow-up, characterized by the fear of disease recurrence or metastases and risk of death.

These results are also evident in each group, in-person evaluation and tele-consult. In the present analysis, patients followed by tele-visits reported comparable quantitative results in terms of depression and post-traumatic stress (DT and IES-R) with respect to the in-person visits group, with the only difference being in terms of anxiety (*p* 0.002). In fact, patients who were followed up with the on-site approach reported a better decrease in the anxiety score. Three main reasons could be hypothesized:

1. First of all, in the TC group, a predominance of poor-prognosis diseases was present (brain tumor, metastatic tumor and head and neck tumor), with a high incidence of recurrence post-treatment and a subsequent higher anxiety score.
2. In the OS group, most of the patients had breast cancer (young patients with good prognosis); the high level of anxiety (Figure 2) was also reduced during follow-up because no recurrence was reported.
3. For phycologists, the evaluation of expression throughout the body is more difficult to evaluate during tele-consults; thus, the psychological strategy could be longer and less precise. The present data are in line with a recent Cochrane publication. The predominance of telephone-delivered interventions for psychological symptoms is unsurprising. Telephone counseling has been shown to be effective in reducing psychological symptoms, including depression and anxiety, in patient populations other than those affected by cancer. Further, this review has demonstrated that telephone-delivered interventions are being developed for managing a range of physical as well as psychological cancer-related symptoms [38].

Surely, our data have some limitations, including small sample size, no randomization, short follow-up, and so on, but are interesting both for underlining the important role of psycho-oncology for oncological patients and the importance of following up with patients by telemedicine.

## 5. Conclusions

As reported in the literature, the data reveal the importance of psychological support for oncological patients [8,39]. For psychological follow-up, telephone interventions, or tele-visits, are convenient for patients, their families and healthcare workers, as they reduce distress and anxiety and activate a propositive behavior. However, considering the absence of solid data, rigorous research on this topic with more homogeneous populations (according to disease status, age, psychological attitude, etc.) and therapeutic approaches, in a greater sample size, are necessary.

## Figures and Tables

**Table 1:** The main characteristics of the whole population study.

Total Number of Patients (pts)	82
Group OS	30 pts (36.6%)
Median age (range)	51 years (35–67)
Sex (male:female)	5:25
Type of cancer	
Breast	18
Prostate	2
Gynecological	3
Others	7
Median RT session (range)	21 (5–30)
Median psycho-therapeutic meetings (range)	3.5 (2–5)
Median psycho-therapeutic meetings in FUP (range)	8 (4–28)
Group TC	52 pts (63.4%)
Median age (range)	48.5 years (18–75)
Sex (male:female)	16:36
Type of cancer	
Breast	21
Brain	10
Prostate	4
Gynecological	5
Others	12
Median RT session (range)	23 (10–33)
Median psycho-therapeutic meetings (range)	4 (2–6)
Median psycho-therapeutic meetings in FUP (range)	8 (4–24)
RT: radiotherapy; OS: on-site; TC: tele-consult	

## Data Availability

Data may be requested from the corresponding author.
